# Queens stay, workers leave: caste-specific responses to fatal infections in an ant

**DOI:** 10.1186/s12862-018-1320-0

**Published:** 2018-12-27

**Authors:** Julia Giehr, Jürgen Heinze

**Affiliations:** Department of Zoology/ Evolutionary Biology, University of Regensburg, 93053 Regensburg, Germany

**Keywords:** Altruism, self-removal, lethal infection, disease management, Terminal investment, Ants, *Temnothorax*

## Abstract

**Background:**

The intense interactions among closely related individuals in animal societies provide perfect conditions for the spread of pathogens. Social insects have therefore evolved counter-measures on the cellular, individual, and social level to reduce the infection risk. One striking example is altruistic self-removal, i.e., lethally infected workers leave the nest and die in isolation to prevent the spread of a contagious disease to their nestmates. Because reproductive queens and egg-laying workers behave less altruistically than non-laying workers, e.g., when it comes to colony defense, we wondered whether moribund egg-layers would show the same self-removal as non-reproductive workers. Furthermore, we investigated how a lethal infection affects reproduction and studied if queens and egg-laying workers intensify their reproductive efforts when their residual reproductive value decreases (“terminal investment”).

**Results:**

We treated queens, egg-laying workers from queenless colonies, and non-laying workers from queenright colonies of the monogynous (single-queened) ant *Temnothorax crassispinus* either with a control solution or a solution containing spores of the entomopathogenic fungus *Metarhizium brunneum*. Lethally infected workers left the nest and died away from it, regardless of their reproductive status. In contrast, infected queens never left the nest and were removed by workers only after they had died. The reproductive investment of queens strongly decreased after the treatment with both, the control solution and the *Metarhizium brunneum* suspension. The egg laying rate in queenless colonies was initially reduced in infected colonies but not in control colonies. Egg number increased again with decreasing number of infected workers.

**Conclusions:**

Queens and workers of the ant *Temnothorax crassispinus* differ in their reaction to an infection risk and a reduced life expectancy. Workers isolate themselves to prevent contagion inside the colony, whereas queens stay in the nest. We did not find terminal investment; instead it appeared that egg-layers completely shut down egg production in response to the lethal infection. Workers in queenless colonies resumed reproduction only after all infected individuals had died, probably again to minimize the risk of infecting the offspring.

**Electronic supplementary material:**

The online version of this article (10.1186/s12862-018-1320-0) contains supplementary material, which is available to authorized users.

## Background

Social insects, such as honeybees, ants, and termites, provide the prime examples of altruistic, self-sacrificing behavior in nature [1], but not all members of the insect society are equally prone to sacrifice themselves. Workers typically do not produce offspring and increase their fitness indirectly through increasing the reproductive output of the queen. They readily engage in costly or dangerous tasks, such as foraging and defense, while the queens focus on laying eggs in the safety of their nests [2–4]. In terms of inclusive fitness [5], the life of an individual worker therefore counts less for the colony than that of the egg-laying queen. This is illustrated, for example, by honey bee workers killing themselves when defending the hive, because their barbed stingers cannot be withdrawn from mammal skin. In contrast, honey bee queens have a smoother stinger with smaller barbs and in principle can use it repeatedly [6]. Similarly, soldiers of *Globitermes* termites and workers of *Colobopsis saundersi* ants “explode” and cover attackers with sticky secretions [7, 8], but such “autothysis” has never been observed in queens. Finally, individuals likely to take over reproduction and gain direct fitness in the future avoid risky tasks, such as colony defense [9–12].

Here, we examine whether queens and workers also differ in the readiness for altruism in their response to lethal infection. Insect societies, in which closely related individuals constantly interact with one another in the confined space of a shared nest, provide ideal conditions for the transmission of pathogens [13]. Therefore, social insects have evolved powerful mechanisms to counteract the transmission of pathogens, including the destruction of infected brood, intense allogrooming, avoidance, killing, and even the walling-in of diseased nestmates [14–16].

One particularly striking behavior is altruistic self-removal: moribund workers of several social insects leave their nests to die outside in isolation, probably preventing the spread of a potentially dangerous pathogen to their nestmates [17–19]. In contrast, dying queens have been observed to stay in the nest, where their corpses may be groomed by workers for days or even weeks (e.g., *Atta mexicana* [20], *Solenopsis invicta* [21], *Pogonomyrmex badius* [22]). As the survival of the colony depends on the queen, it might more strongly invest into its immune defense than workers [23–25] and try to overcome an infection. Alternatively, fatally infected queens might turn all available resources into reproduction in the nest, resulting in terminal investment [26, 27]. Indeed, *Cardiocondyla obscurior* ant queens dying from an infection increased their egg laying before imminent death [28], whereas a non-lethal injury led to the upregulation of immune genes and a temporary decline of egg laying rate [25].

Unfortunately, the behavior of dying queens and workers has never been studied in the same species, which makes it difficult to determine whether the response to pathogen stress and impending death is caste-specific or merely varies among species. Here, we infected workers and queens of the monogynous (single-queened) ant *Temnothorax crassispinus* with spores of the entomopathogenic fungus *Metarhizium brunneum*. We investigated the behavior of conspecific dying queens and workers in queenright colonies and also queenless colony fragments. This set up enabled us to examine the specific effects of both caste (queens vs. workers) and reproductive status (reproductive vs. non-reproductive individuals), as in queenless colonies a small number of socially dominant *T. crassispinus* workers may lay eggs [29, 30]. From the above-cited observations [17–22] we expected fatally infected, non-reproductive workers from both queenless and queenright colonies to die in isolation outside the nest but queens and egg-laying workers to die inside.

The results of our study indicate caste-specific behavior of queens and workers dying from an infection: all moribund queens remained in the nest and all infected workers, both reproductive and non-reproductive, left the nest and died in isolation. Furthermore, in contrast to our expectation that egg-laying rates increase after exposure to a lethal pathogen (see *Cardiocondyla obscurior* queens [28]), egg-laying by queens and workers stopped after *Metarhizium* treatment and egg numbers slowly began to increase again in queenless colonies only after all or at least most of the infected nestmates had died.

## Methods

*Temnothorax crassispinus* is a small monogynous, monandrous ant species (a single, singly-mated queen per colony), which lives in small colonies of up to 300 individuals in hollow acorns or twigs throughout Eastern Central Europe [30–33]. Colonies were collected in July 2017 in deciduous forests around Regensburg, Germany. We split 19 colonies into queenright (QR) and queenless (QL) parts consisting each of 18 old (darkly colored) and 18 young (lightly colored) workers or soon to emerge worker pupae and, in QR colonies, one queen. After queen loss, young workers engage in dominance interactions and one or a few dominant workers begin to produce male offspring from unfertilized eggs after one to 4 weeks [29, 34]. Old workers are needed for the successful establishment of split colonies in the laboratory as they conduct non-reproductive tasks before young workers are old enough to take over.

To distinguish old workers from aging young workers, we marked all old workers by clipping the tarsae of the middle leg. Young workers remained unharmed. Colonies were reared in small plastic boxes (10x10x3 cm^3^) with a moistened plaster floor in incubators under 23 °C / 15 °C day / night cycles. They were fed twice per week with cockroaches and sugar solution. Dead individuals were removed but not replaced as this would have disturbed the established dominance hierarchies [34]. Hence, worker numbers varied slightly among colonies.

Four weeks after the experimental colonies were set up we counted and removed all brood. We then noted the egg number daily for 10 days to estimate the reproductive rate of each colony before the treatment. Thereafter, all individuals were censused again and all brood was removed. To investigate whether queens and / or young, egg-laying workers adjust their behavior and reproductive efforts to pathogen stress, we treated the queen / all young workers with either a control solution (0.05% Triton X) or a *Metarhizium brunneum* spores suspension (1 × 10^8 spores / ml 0.05% Triton X). Preliminary tests had shown that 71% of *T. crassispinus* workers develop a lethal infection within 10 days after being dipped for 1 s into 500 μl of a spore suspension of this concentration. *M. brunneum* is an obligate-killing entomopathogenic fungus that penetrates the host cuticle within 48 h after exposure [35, 36]. Subsequent hyphal growth and the release of toxins result in the death of the host. The fungus completes its life cycle by producing infectious conidiospores on the host surface [35–38].

Our experimental setup consisted of five different treatments:

1. queenless control (*n* = 9): all young workers treated with Triton X;
2. queenless infected (*n* = 10): all young workers treated with spore solution;
3. queenright control (*n* = 6): all young workers and queen treated with Triton X;
4. queenright, workers infected (*n* = 5): all young workers treated with spore solution, queen treated with Triton X;
5. queenright, queen infected (*n* = 8): all young workers treated with 0.05% Triton X, queen treated with spore solution.

We treated all young workers and the queen if present with either the control or spore solution. Note that in most colonies a few young workers disappeared from the nest or died between set-up and treatment so that the number of these individuals is typically lower than 18 per colony (see results).

The marked old workers remained completely untreated. After exposure all individuals were placed on sterilized filter papers to remove surplus fluid and thereafter were kept in groups isolated from their colonies for 36 to 40 h to inhibit immediate transmission of spores to their uninfected and untreated nestmates. This short period of isolation is not long enough to change colony odor or the dominance hierarchy in the colony, and none of the returned workers or queens were attacked after being placed back into the nest. After return to their colonies, we noted the position and condition of queens and workers every morning and counted the numbers of eggs and dead individuals once every 24 h for the first 10 days and subsequently three times a week. Sampling was conducted blindly regarding the control and experimental groups.

All dead individuals were removed. To verify that the ants had died of an infection with *M. brunneum* their corpses were immersed for 5 s in 70% EtOH, rinsed with distilled water, and surface-sterilized for 1 min in 1% NaClO. Subsequently, they were again cleaned with distilled water and dried on filter paper. The gaster was removed with sterile forceps and frozen at − 20 °C for ovary dissections (see below). The head and thorax were placed into a sterilized Petri dish containing a moist cotton ball and lined with moist filter paper. After covering the Petri dish with a lid, the dish was sealed with Parafilm to prevent the loss of humidity. Samples were checked regularly until spore growth was visible on the ant surface or for a maximum of 3 weeks. The reproductive status of workers from both queenless and queenright colonies was determined by dissecting the ovaries of 70 workers that had died outside the nest and whose corpses had been frozen within ≤24 h after death (41 infected and 9 uninfected young workers, 20 uninfected old workers).

As only few eggs were laid by the colonies during the first weeks after treatment, we increased the temperature in the incubators to 26 °C/22 °C (day/night) on day 39 to accelerate egg production. Individuals that died later were similarly sterilized and checked for spore growth. Four control workers could not be used for surface sterilization due to an advanced decay. Hence, the sample size differs in this case from that of the survival analysis. The final analysis of death rate was conducted after 75 days if not stated otherwise. For egg number comparisons day zero was defined as the day of the return of the treated individuals to their colonies, while for survival analysis day zero was the day of the treatment. Data (Additional file 1) were analyzed with R 3.2.3 software (R Development Core Team) using packages “vegan” for PerMANOVA [39] and “survival” [40] for the Kaplan–Meier survival analysis and graph. Pairwise survival comparisons were conducted using the package “survminer” [41]. In addition to the treatment group as predictor we included the colony as a random effect (“frailty”) in the Cox survival analysis model [40] of the young workers to control for survival differences between colonies. Data from surviving individuals were included as censored. Kruskal-Wallis tests were used for group comparisons, Mann-Whitney U-test (unpaired) and Wilcoxon signed-rank test (paired) for two-sample comparisons. All pairwise tests were corrected for multiple testing according to a false discovery rate (p adjust method: “fdr”) [42].

## Results

### Survival rate of treated workers and queens

**Fig. 1:**
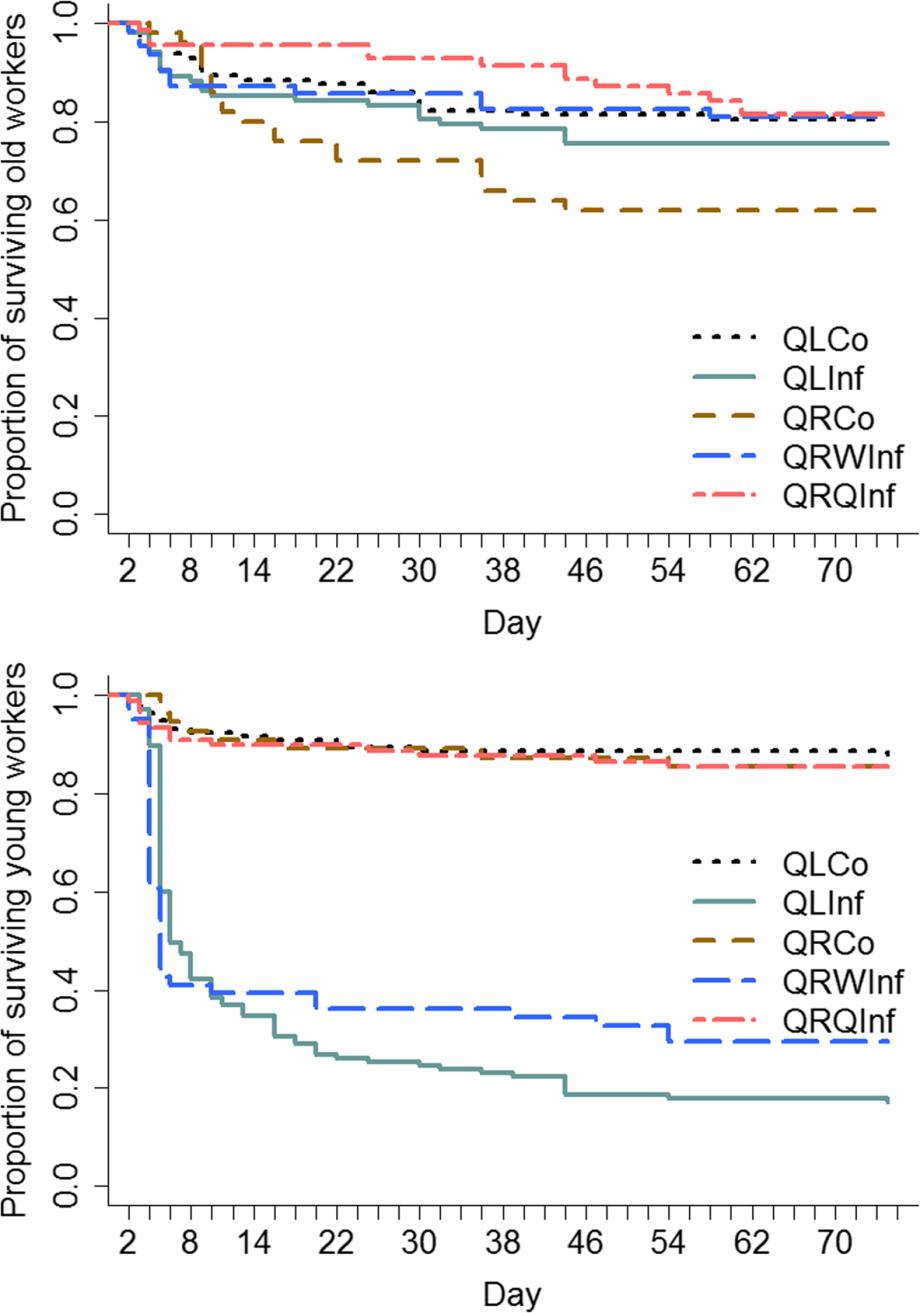
Survival of workers of the ant *Temnothorax crassispinus* was significantly decreased in young workers suffering an infection of the entomopathogenic fungus *Metarhizium brunneum* (QLInf, QRWInf) compared to young workers treated with a control solution (QLCo, QRCo, QRQInf) in queenless (QL) and queenright (QR) colonies. Old, untreated control workers did not show a reduction in lifespan between treatment groups. The experiment was terminated 75 days after the treatment and the lifespans of surviving workers were included as censored data. A colony-wise comparison also shows significant differences in survival rates between infected and uninfected individuals (see main text)

At the day of the treatment each colony fragment contained 12.5 ± 2.7 (mean ± sd) young workers and 10.5 ± 3.3 (mean ± sd) old workers. Direct spore contact strongly reduced the lifespan of both queens and workers: 158 of 196 young workers (median percentage dead workers per colony 85%; Q1 67%; Q3 96%) and six of eight queens died within 75 days after treatment with spore solution, in contrast to 33 of 266 young control workers (median percentage dead workers per colony 9%; Q1 7%; Q3 14%; Mann-Whitney U-test: U = 330, *p* < 0.0001) and one of 11 queens (Fisher’s exact test, *p* < 0.0063) exposed to Triton X and 91 of 398 old workers (median percentage dead workers per colony 18%; Q1 10%; Q3 33%) that had not been treated at all. Of the 158 dead, spore-treated workers, 23 (14.5%, eight QL and one QR colony) did not produce any *M. brunneum* spores after surface sterilization. Four of them did not show any pathogen load and 19 produced spores of other, unidentified pathogens. One of 33 (3%, one QR colony) of the young control workers that had died during the experiment was infected with *M. brunneum*. Of the 91 dead, untreated old workers, 11 (12%, two QL and 5 QR colonies) produced *M. brunneum* spores, 70 (77%) produced spores of unidentified pathogens, and 10 (11%, 4 QL and 4 QR colonies) did not show any pathogenic growth. Old workers infected with *M. brunneum* were excluded from survival analysis as these resulted from an uncontrolled infection by nestmates. Whereas across the different treatment groups untreated old workers did not differ in lifespan (χ^2^ = 8.7, df = 4, *p* = 0.068, Fig. 1), young spore-exposed workers showed a strongly decreased survival compared to young workers treated with the control solution (χ^2^ = 238, df = 4, *p* < 0.0001, for details see Additional file 2: Table S1). In addition to the treatment effect we also observed that colonies were differently sensitive to pathogen exposure (treatment: χ^2^ = 47.56, df = 1.0, *p* < 0.0001, colony: χ^2^ = 27.02, df = 8.8, *p* = 0.0012). The percentage of infected workers still alive 10 days after exposure varied significantly among colonies (χ^2^ = 41.8, df = 9, *p* < 0.0001, for details see Additional file 2: Table S2). There was no colony effect on survival in young untreated control workers (χ^2^ = 6.7, df = 8, *p* = 0.57). The presence of an uninfected queen did not have any effect on worker lifespan (QRWInf vs. QLInf: *p* = 0.740, see Additional file 2: Table S1). The survival rate of spore-exposed queens was also strongly reduced (χ^2^ = 8.6, df = 2, *p* = 0.013; survival (days): 5, 6, 6, 7, 10, 10, > 74, > 74).

### Behavior of dying workers and queens

*Metarhizium*-treated individuals were not prevented from entering the nest or from approaching healthy individuals or brood, e.g., infected and uninfected nestmates did not separate. One infected worker was observed carrying an egg. During the first 10 days after treatment the proportion of workers observed outside the nest was significantly higher in colonies in which workers were infected (old workers, control colonies: median 0.017; Q1 0.013 Q3 0.022; old workers, infected colonies: median 0.060; Q1 0.041; Q3 0.079; Mann-Whitney U-test, U = 71, *p* = 0.0113, young workers, control colonies: median 0.008; Q1 0.000; Q3 0.011; young workers, infected colonies: median 0.034; Q1 0.021; Q3 0.042; U = 66, *p* = 0.026, Additional file 2: Figure S1). Additionally, in colonies with infected workers the number of dead young workers was significantly correlated with the number of young workers observed outside the nest on the previous day (Spearman rank tests, *n* = 11, 0.098 < r_s_ < 1, mean 0.590, 0.0001 < *p* < 0.802, Fisher’s combined probability, df = 22, χ^2^ = 106.110, *p* < 0.0001) but not so in old workers (*n* = 9, − 0.188 < r_s_ < 0.546, mean 0.183, 0.129 < *p* < 0.889, Fisher’s combined probability, df = 18, χ^2^ = 14.183, *p* = 0.717). Both observations suggest that dying workers leave the nest, as described previously in a related species [17]. Furthermore, we directly observed 38 *M. brunneum*-infected workers dying outside – they were easily identified by their cramped body posture and stiff locomotion when touched with forceps. In four cases in three different *Metarhizium*-treated colonies we observed untreated, old workers outside the nest carrying the corpse of a young worker. Such behavior was never observed in control colonies.

We could not systematically test the reproductive state of deceased workers because their ovaries were rapidly destroyed by intense hyphal growth. Nevertheless, dissections revealed that of all the examined workers that had died outside the nest, 27% had eggs in development (for details see below). Furthermore, direct observations of egg laying by four workers that died after spore exposure and the presence of an egg laid in isolation by one of 18 infected workers, which later all died, suggests that several of the workers that had died outside had been reproductive. In conclusion, both non-reproductive and reproductive workers left the nest to die outside.

In contrast, queens were never observed alive outside the nest chamber and while all 158 lethally infected workers, including the egg layers, left the nest before death, the six moribund queens died in the nest (Fisher’s exact test, *p* < 0.0001). Three of six infected dead queens, but none of the infected dead workers, showed mutilations of legs and / or antennae. One infected queen was observed lying motionlessly in a stiff posture inside the nest and 1 day later was found dead outside the nest. The corpse of another infected queen was carried outside the nest by an untreated old worker. One queen, treated with Triton X, was found decapitated outside the nest 4 days after treatment. Ovary dissection revealed that the ovaries of the latter queen were undeveloped and that its spermatheca was empty, indicating that it had not been reproductive. This colony was excluded from further analyses.

### Effect of pathogen-exposure on worker and queen fecundity

Egg numbers produced before and after treatment were analyzed separately for queenless and queenright colonies, as infected queens died very rapidly while egg production in queenless colonies could be monitored over much longer periods. Workers typically do not become reproductive in the presence of the queen and both treatment and queen presence had a strong effect on eggs present 3 days before and 3 days after the treatment (PerMANOVA; treatment: *F* = 2.8, df = 2, *p* = 0.038; time: *F* = 7.6, df = 1, *p* = 0.0024; queen presence: *F* = 10.2, df = 1, *p* = 0.0007).

**Fig. 2:**
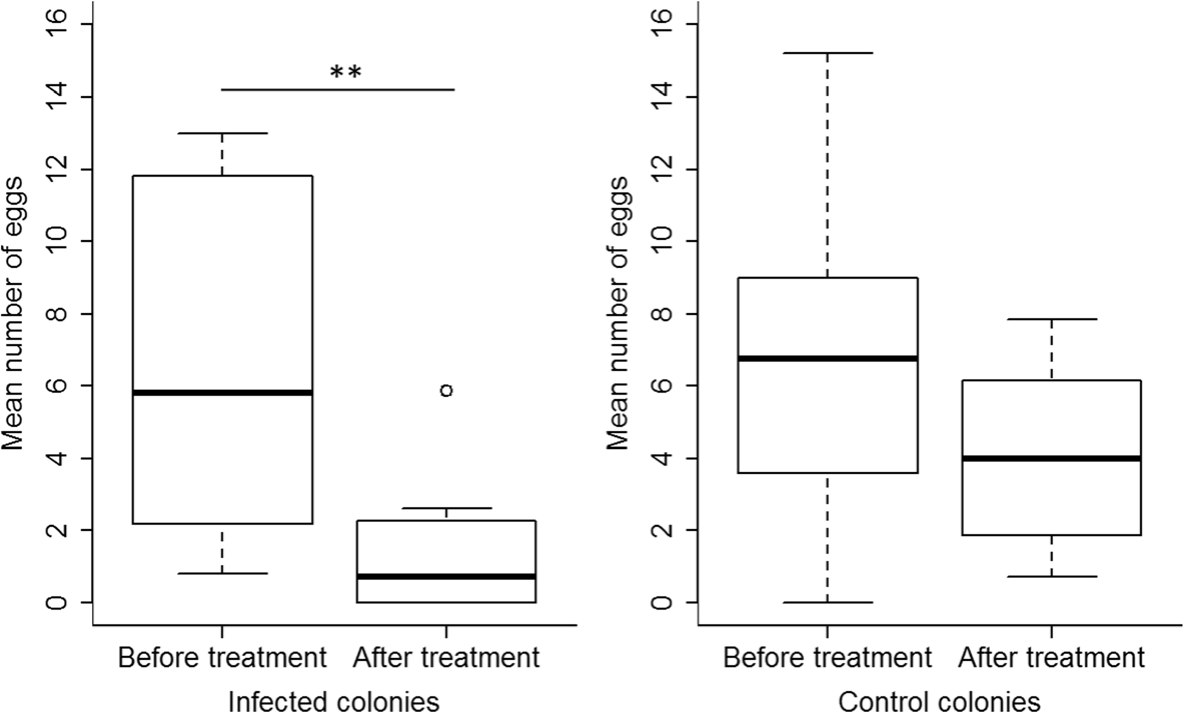
Mean number of eggs produced in queenless colonies of *Temnothorax crassispinus* during 7 days before and after the treatment. Workers infected with *Metarhizium brunneum* (left) laid significantly fewer eggs than workers of the control group (right) after, but not before the treatment. Boxplots show medians, 25 and 75 quartiles, and 95% percentiles (** *p* < 0.01 corrected for a false discovery rate: “fdr”)

In queenless colonies, the reproductive rates did not differ between control and infected colonies during 7 days before the treatment (Mann-Whitney U-test: U = 43, *p* = 0.903), but differed at marginal significance thereafter (U = 73, *p* = 0.052). Whereas the weekly egg laying rate did not change in the control group (Wilcoxon signed rank test: V = 33, *p* = 0.250), it decreased after infection (V = 55, corrected *p*-value = 0.0039, Fig. 2). The number of produced eggs was significantly affected by infection independent of the worker number inside the nest (PerMANOVA; treatment: df = 1, *F* = 3.6, *p* = 0.049; number of young workers: df = 1, *F* = 0.8, *p* = 0.477; total number of workers: df = 1, *F* = 0.35 *p* = 0.745; treatment * number of young workers: df = 1, *F* = 2.5, *p* = 0.083).

All queenless control colonies had contained eggs before the treatment, but in only six of ten queenless infected colonies workers continued to lay eggs after the treatment (see Table 1). Furthermore, infected colonies did not only produce fewer eggs after the treatment, but as long as infected workers were present the eggs produced in the colonies frequently disappeared (Additional file 2: Figure S2). The presence of eggs was affected by the number of infected individuals and its interaction with the day of the experiment. The number of infected individuals decreased with time and there was also a colony effect (PerMANOVA; number of infected individuals: df = 1, *F* = 3.9, *p* = 0.035; day: df = 25, *F* = 3.08, *p* = 0.0001; colony: df = 9, *F* = 9.14, *p* = 0.0001; number of infected individuals*day: df = 25, *F* = 1.8, *p* = 0.004).

**Table 1:** Number of queenless (QL) and queenright (QR) colonies of the ant *Temnothorax crassispinus* in the different treatment groups containing no eggs during the time before treatment (BT) and after the treatment (AT) (control workers: QLCo, QRCo, QRQInf; infected workers: QLInf, QRWinf; infected queen: QRQInf). In one QRCo colony all workers except one died during the experimental period and this colony was excluded from the 10 weeks after treatment analysis

Queenless	Before Treatment (without/total)	7 days AT (without/total)	10 weeks AT (without/total)
QLCo	0/9	0/9	0/9
QLInf	0/10	4/10	2/10
Queenright	Before treatment (without/total)	After Treatment (without/total)	10 weeks AT (without/total)
QRCo	0/5	4/5	1/4
QRWInf	1/5	4/5	2/5
QRQInf	3/8	7/8	2/2 (with queen) 0/6 (queenless)

**Fig. 3:**
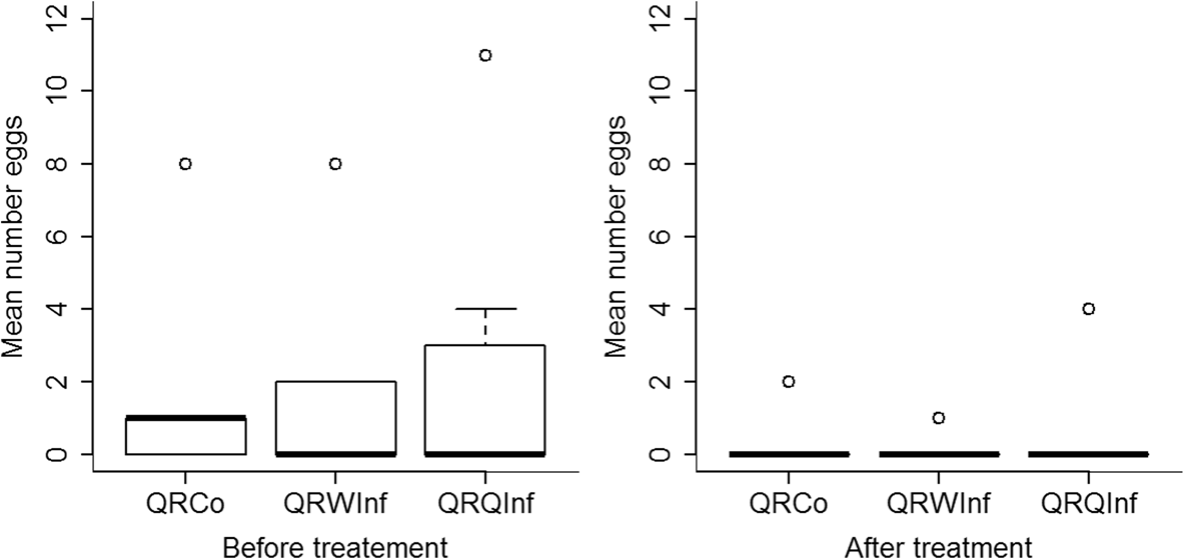
Mean number of eggs produced in queenright colonies of *Temnothorax crassispinus* during 3 days before (left) and after (right) the treatment. Both groups decreased their egg laying rate. The reproductive rate of queens infected with *Metarhizium brunneum* (QRQInf) neither differed from that of control queens (QRCo, QRWInf) before nor after the treatment. The treatment itself seems to result in a reproductive shutdown in queens of all treatment groups. Boxplots show medians, 25 and 75 quartiles, and 95% percentiles

Queens did not lay any eggs during isolation and egg production in queenright colonies could only be analyzed for the first 3 days after return to the colony, as the first queen had already died on the third day. The median egg number produced during 3 days did neither differ among queens before (χ^2^ = 0.1, df = 2, *p* = 0.948) nor after the treatment (χ^2^ = 0.08, df = 2, *p* = 0.959, Fig. 3). Queens appeared to sensitively react to the treatment with an almost complete reproductive shut-down regardless of whether they had been exposed to spores or only Triton-X (PerMANOVA; treatment: df = 1, *F* = 0.15, *p* = 0.957; number of young workers: df = 1, *F* = 0.8, *p* = 0.395; total number of workers: df = 1, *F* = 0.03, *p* = 0.956; treatment * number of young workers: df = 4, *F* = 0.62, *p* = 0.556). Although all queenright colonies had contained eggs before the experiment started, no new eggs had appeared in four of 18 colonies even before the treatment. After the treatment, no eggs were laid in seven of eight colonies with an infected queen and four of five colonies each with an uninfected queen and either infected or Triton X treated workers (analyzed per colony until the death of the queen; Fisher’s exact test, *p* = 0.0006). Although queens reacted sensitively to the treatment itself, they also appeared to be capable of adjusting their reproductive rate to the presence of infected workers, as queens of the control group produced more eggs after treatment than control queens with infected workers (PerMANOVA: treatment: df = 1, *F* = 7.2, *p* = 0.0063, day of the experiment: df = 23, *F* = 1.6, *p* = 0.03, colony: df = 8, *F* = 7.4, *p* = 0.0001, see Additional file 2: Figure S3).

Interestingly, workers did not start to reproduce in the presence of the queen even when the latter refrained from laying eggs. Six of eight queens died within 10 days after spore contact but workers began to continuously produce eggs in the formerly queenright nests only 37 to 59 days after the queen’s death (*n* = 4, median 40.5). In two additional colonies eggs were sporadically produced but vanished even after 78 days. The initial presence of an infected queen appeared to suppress and delay worker reproduction compared to the removal of a healthy queen (median 17 days, range 6 to 35 days; see also [29]). At the end of the experiment still no eggs were present in the nests of the two surviving infected queens. Colonies, in which the queen had died, appeared to produce more eggs than control queenright colonies (Mann-Whitney U-test: U = 4, *p* = 0.053, QRQInf *n* = 6, median 19, Q1 4.75, Q3 44.5; QRCo *n* = 5, median = 0, Q1 0, Q3 4; one colony with an uninfected but deceased queen in QRCo and two colonies with a still living, infected queen QRQInf were excluded from the analysis).

**Fig. 4:**
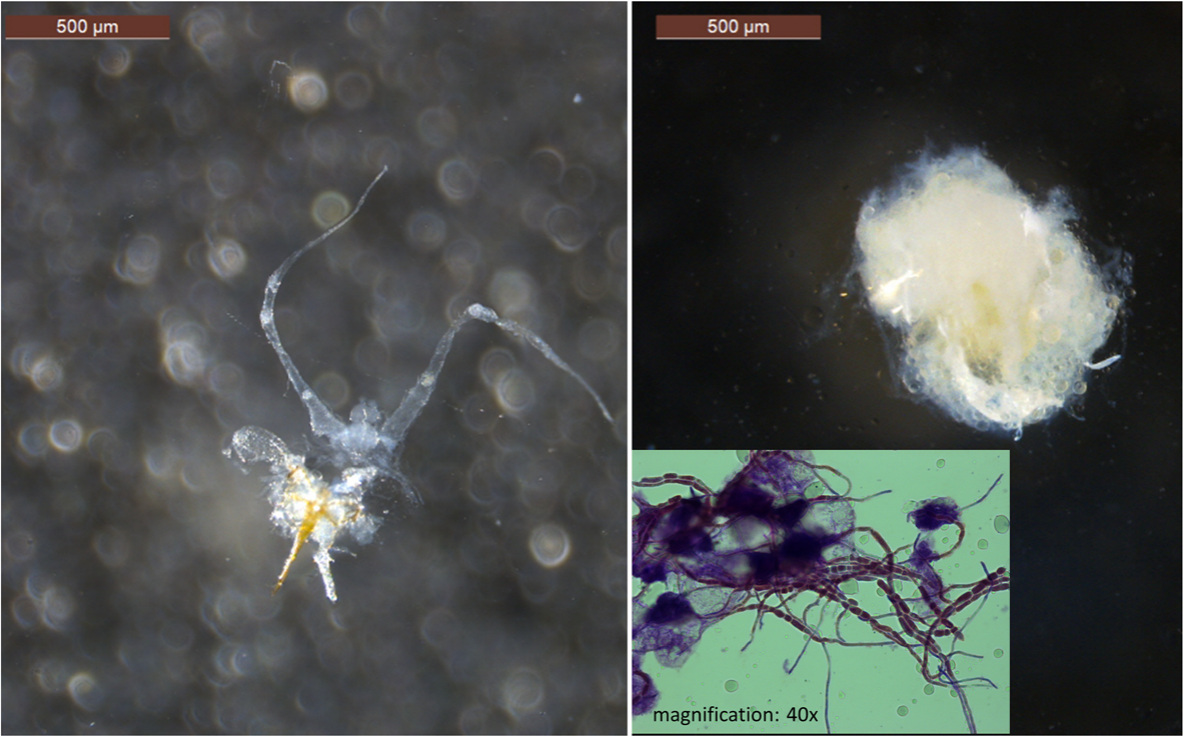
Ovaries of control (left, death 2 days after treatment of colony members) and *Metarhizium-*infected (right, death 4 days after treatment) *Temnothorax crassispinus* workers*.* The gaster contents of infected workers are unidentifiable as fungal hyphae have spread throughout the gaster (see microscope picture at the bottom right; magnification 40x), whereas the ovaries of the uninfected workers are clearly visible

The preparation of the ovaries of dead workers showed intense hyphal growth in the gasters of all workers with spore growth after surface sterilization (see Fig. 4). In 27 of 41 infected workers, internal organs, especially the ovaries, were no longer visible and it was impossible to determine their reproductive status. The ovaries of nine workers (64%, four QL and three QR colonies) had at least one egg in development, while the ovaries of five workers appeared to be undeveloped. In contrast, the ovaries of nine uninfected young workers and 20 uninfected old workers were clearly visible and differed in developmental status. One of nine uninfected young workers (11%, one QR colony) and nine of 20 (45%, one worker each in four QR colonies and three QL colonies and two workers in one QR colony) untreated old workers had one or two eggs in development. Traces of previous egg laying (e.g., corpora lutea and / or developing eggs) were found in one infected, dead worker each from two colonies with an uninfected queen.

Dissections of the queens revealed hyphal growth throughout the gasters of five of six infected queens. Ovaries and corpora lutea could be detected in only one of the five queens; in the sixth queen, ovaries and corpora lutea were still visible, but no maturing eggs were present (see Additional file 2: Figure S4).

## Discussion

The intensive social contact among closely related individuals in densely crowded nests makes insect societies particularly vulnerable to contagious diseases [13, 43]. Social insects were successful in evolution because they evolved a particularly sophisticated multi-layer defense against pathogens and parasites, ranging from cellular to individual and collective responses [44–53]. Behavioral responses, such as intensive self-grooming, nest cleaning, self-medication (e.g., [46, 54, 55]), and self-removal [17, 18] have as yet mostly been studied in the worker caste and except for changes in physiology and reproduction (e.g., [56–59]) little is known about how queens respond to an infection.

We here report that queens and workers of the ant *Temnothorax crassispinus* react differently to an infection with the obligatorily killing pathogen *Metarhizium brunneum*: while moribund workers, regardless of ovarian status, left the nest to die away from it, queens stayed and were carried out of the nest only after their death. Our study shows that the altruistic self-removal is a caste-specific behavior and does not vary with the reproductive status of the workers. Furthermore, the presence of infected nestmates was associated with a strong decrease of egg laying even by uninfected nestmates.

Through altruistic self-removal [17, 18] and the withdrawal from social interactions [19] infected workers certainly minimize the risk of transmitting a pathogen to brood and adult nestmates. Altruistic self-removal is not caused by a specific manipulation of the pathogen but is induced by the workers to isolate themselves from other colony members to prevent possible risks for nestmates as previously shown for moribund workers by Heinze and Walter [17]. Horizontal transmission of spores has been documented in several insects (e.g., [60–62]) and cross-infection may also have been the cause of old, untreated workers dying from an infection with this fungus, in particular as old workers were seen handling corpses. It is therefore easy to see why infected workers leave the nest to die isolated from their nestmates.

Considering the social withdrawal and pathogen control in workers, it is more difficult to understand why infected queens stayed and were removed by workers only after they had died. Even a single-queened colony should benefit from the self-removal or the early expulsion of a fatally infected queen. However, compared to workers most social insect queens have a longer lifespan and survive better under stressful conditions [63–65]. They might also have a higher chance to survive an infection than workers by investing more in immune defense ([23, 24]; see also below). Queens therefore might remain in the nest as long as there is a chance to recover. Furthermore, Rueppell and colleagues showed that while founding queens of *Temnothorax* are capable of conducting worker tasks and leave the nest to forage, they lose this behavioral plasticity in established colonies and remain in the nest even when workers are removed [66] whereas reproductive workers may still be capable of conducting non-reproductive tasks and leave the nest (e.g., [67]). Hence, fatally infected queens might simply not have been capable of leaving the nest independently. Workers apparently do not discriminate against infected nestmates before the fungus has begun to produce spores on the cadaver of its host (e.g., [68]) and therefore could reduce the contagion risk for the colony only after the queen’s death by removing its corpse [20, 69, 70].

Both control and spore-treated queens refrained from reproduction for several weeks after treatment and even 10 weeks later only half of them had recommenced to lay eggs. A reduction of reproductive efforts has previously been observed in honey bee queens infected with the fungus-related pathogen *Nosema apis* [57] and *Metarhizium*-infected queens of the ant *Lasius niger* [69]. In the latter this was suggested to result from increased investment in the immune system, similar to the temporary drop of egg laying rates following an injury in the ant *Cardiocondyla obscurior* [25]. Since *Temnothorax* queens can live for several years [71], after a potentially dangerous treatment they might invest more strongly in pathogen defense and the restoration of their body condition than in reproduction. Even the contact with the solvent, handling stress, or the absence of allogrooming and trophallaxis during the isolation phase affected the physiology of queens in a way that they stopped egg laying. In addition, queens appeared to react sensitively to the presence of infected workers by reducing their reproductive efforts.

While a few *Temnothorax* workers quickly begin to lay eggs when the queen is removed from the colony [29, 30, 34, 72], no worker egg laying was observed in the presence of a non-laying, infected queen. This supports the view that that the stop of egg laying does not necessarily mean the loss of queen control (see also [73] for social dominance of ovariectomized wasp queens). Similarly, only few eggs were laid in queenless colonies after workers had been infected. Freshly laid eggs quickly disappeared, and egg numbers increased only after most of the infected nestmates had died, probably to prevent the cross-infection of newly produced brood.

Although *Metarhizium* infection strongly decreased the survival of queens and laying workers we could not observe terminal investment [26, 27], in contrast to what we have previously reported for *Cardiocondyla* ant queens [28]. This discrepancy might reflect the different life history of the two species. The single queens of many *Temnothorax* colonies may live for 10 years and longer [71], while queens in the multi-queen colonies of *C. obscurior* are short-lived (mean: 26 weeks [74]) and can quickly be replaced by female sexuals, which after eclosing from the brood mate with their brothers in the natal nest [75]. *Temnothorax* queens might therefore preferentially invest in individual and colony immunity as their future reproductive success depends more strongly on their survival than in *Cardiocondyla*. Further studies are needed to investigate how the life span of ant queens is associated with their immune investment.

## Conclusions

Our data show that workers and queens of the ant *Temnothorax crassispinus* react differently to infection with the entomopathogenic fungus *Metarhizium brunneum.* Infected queens stayed inside the nest but refrained from reproduction. Workers, independent of their reproductive state, left the nest and died in social isolation. Both, queens and workers reduced reproductive investment after the treatment with *M. brunneum.* Egg numbers increased with the decreasing number of infected individuals in queenless colonies, but workers did not lay eggs in the presence of the queen, even when the queen was sick and did not reproduce. Our study reports for the first time a caste-specific behavior in response to lethal infections in the same species.

## Additional files

Additional file 1: Datasets supporting the article. (XLSX 61 kb)

Additional file 2: **Table S1.** Pairwise survival comparison of young *Temnothorax crassispinus* workers in queenless (QL) and queenright (QR) colonies, treated with a control solution (QLCo, QRCo, QRQInf) or infected with *Metarhizium brunneu*, (QLInf, QRWInf). Significant *p*-values (corrected for a false discovery rate “fdr”) are marked in bold. **Table S2.** Pairwise survival comparison of young *Temnothorax crassispinus* workers of queenless colonies infected with *Metarhizum brunneum*. Significant *p*-values (corrected for a false discovery rate “fdr”) are marked in bold. **Figure S1.** Proportion of old (left) and young (right) *Temnothorax crassispinus* workers leaving the nest in colonies with young workers either infected with *Metarhizium brunneum* (infected colonies) or treated with a control solution (control colonies) independent of queen presence. Both young and old workers leave the nest more often when they themselves or nestmates are infected. Boxplots show median, 25 and 75 quartile and 95% percentile (*0.05 < *p* > 0.01; corrected for a false discovery rate: “fdr”). **Figure S2.** Reproductive rate of queenless *Temnothorax crassispinus* colonies during the first 25 days after the treatment. Eggs in infected colonies (top) vanish frequently and the colonies produce less eggs than control colonies (bottom). **Figure S3.** Number of eggs produced in queenright *T. crassispinus* control colonies (QRCo, left) or colonies with a control queen and *M. brunneum* infected workers (QRWInf, right). Whereas control queens increase their egg laying rate with time, the small number of eggs produced in colonies with infected workers vanish repeatedly. **Figure S4.** Ovaries of *Temnothorax crassispinus* queens infected with *Metarhizium brunneum*. Developmental status of the ovaries cannot be analyzed in five out of six queens as the gaster show excessive spore growth. (DOCX 2791 kb)

## Abbreviations

QL: queenless
QLCo: queenless control
QLInf: queenless infected
QR: queenright
QRCo: queenright control
QRQInf: queenright queen infected
QRWInf: queenright worker infected
