# Supplementary material for: Queens stay, workers leave: caste-specific responses to fatal infections in an ant
Source: BMC Evol Biol. 2018 Dec 27;18:202. doi: 10.1186/s12862-018-1320-0 (PMC6307282; doi:10.1186/s12862-018-1320-0)
Supplement: Supplementary file 2 — Table S1. Pairwise survival comparison of young Temnothorax crassispinus workers in queenless (QL) and queenright (QR) colonies, treated with a control solution (QLCo, QRCo, QRQInf) or infected with Metarhizium brunneu, (QLInf, QRWInf). Significant p-values (corrected for a false discovery rate “fdr”) are marked in bold. Table S2. Pairwise survival comparison of young Temnothorax crassispinus workers of queenless colonies infected with Metarhizum brunneum. Significant p-values (corrected for a false discovery rate “fdr”) are marked in bold. Figure S1. Proportion of old (left) and young (right) Temnothorax crassispinus workers leaving the nest in colonies with young workers either infected with Metarhizium brunneum (infected colonies) or treated with a control solution (control colonies) independent of queen presence. Both young and old workers leave the nest more often when they themselves or nestmates are infected. Boxplots show median, 25 and 75 quartile and 95% percentile (*0.05 < p > 0.01; corrected for a false discovery rate: “fdr”). Figure S2. Reproductive rate of queenless Temnothorax crassispinus colonies during the first 25 days after the treatment. Eggs in infected colonies (top) vanish frequently and the colonies produce less eggs than control colonies (bottom). Figure S3. Number of eggs produced in queenright T. crassispinus control colonies (QRCo, left) or colonies with a control queen and M. brunneum infected workers (QRWInf, right). Whereas control queens increase their egg laying rate with time, the small number of eggs produced in colonies with infected workers vanish repeatedly. Figure S4. Ovaries of Temnothorax crassispinus queens infected with Metarhizium brunneum. Developmental status of the ovaries cannot be analyzed in five out of six queens as the gaster show excessive spore growth. (DOCX 2791 kb) [file 12862_2018_1320_MOESM2_ESM.docx]

**Electronic Supplemental Material**

# Queens stay, workers leave: caste-specific responses to fatal infections in an ant

# J. Giehr and J. Heinze

University of Regensburg, Biology I, Department of Zoology/Evolutionary Biology, D-93053 Regensburg
Corresponding author: Julia Giehr (Julia.Giehr@ur.de)

Table S1: Pairwise survival comparison of young *Temnothorax crassispinus* workers in queenless (QL) and queenright (QR) colonies, treated with a control solution (QLCo, QRCo, QRQInf) or infected with *Metarhizium brunneu,* (QLInf, QRWInf). Significant p-values (corrected for a false discovery rate “fdr”) are marked in bold.

|  | QLCo | QLInf | QRCo QRWInf |
| --- | --- | --- | --- |
| QLInf | **p < 0.0001** |  | |
| QRCo |  |  |  |
|  | p = 0.74 | **p < 0.0001** |  |
| QRWInf |  |  |  |
|  | **p < 0.0001** | p = 0.74 | **p < 0.0001** |
| QRQInf |  |  |  |
|  | p = 0.74 | **p < 0.0001** | p = 0.95 **p < 0.0001** |

Table S2: Pairwise survival comparison of young *Temnothorax crassispinus* workers of queenless colonies infected with *Metarhizum brunneum*. Significant p-values (corrected for a false discovery rate “fdr”) are marked in bold.

| colony | 1 | | |  |  | 2 |  |  | 3 |  |  | 4 |  |  | 5 |  |  | 6 |  |  | 7 |  |  | 8 |  |  | 9 |
| --- | --- | --- | --- | --- | --- | --- | --- | --- | --- | --- | --- | --- | --- | --- | --- | --- | --- | --- | --- | --- | --- | --- | --- | --- | --- | --- | --- |
| 2 | p | = | 0.149 |  |  |  |  |  |  |  |  |  |  |  |  |  |  |  |  |  |  |  |  |  |  |  |  |
| 3 | p | = | 0.714 | p | = | 0.553 |  |  |  |  |  |  |  |  |  |  |  |  |  |  |  |  |  |  |  |  |  |
| 4  5 | p | = | 0.754 | p | = | 0.177 | p | = | 0.652 |  |  |  |  |  |  |  |  |  |  |  |  |  |  |  |  |  |  |
|  | **p** | **=** | **0.011** | p | = | 0.144 | p | = | 0.0551 | p | = | 0.007 |  |  |  |  |  |  |  |  |  |  |  |  |  |  |  |
| 6  7 | **p** | **=** | **0.007** | **p** | **=** | **0.019** | **p** | **=** | **0.021** | p | = | 0.005 | p | = | 0.304 |  |  |  |  |  |  |  |  |  |  |  |  |
|  | p | = | 0.737 | p | = | 0.442 | p | = | 0.572 | p | = | 0.399 | **p** | **=** | **0.014** | **p** | **=** | **0.005** |  |  |  |  |  |  |  |  |  |
| 8  9 | p | = | 0.252 | p | = | 0.714 | p | = | 0.635 | p | = | 0.384 | p | = | 0.060 | **p** | **=** | **0.012** | p | = | 0.635 |  |  |  |  |  |  |
|  | **p** | **=** | **0.021** | p | = | 0.373 | p | = | 0.111 | p | = | 0.051 | p | = | 0.653 | p | = | 0.149 | p | = | 0.095 | p | = | 0.188 |  |  |  |
| 10 | **p** | **=** | **0.012** | p | = | 0.111 | **p** | **=** | **0.049** | p | = | 0.012 | p | = | 0.694 | p | = | 0.553 | **p** | **=** | **0.013** | p | = | 0.055 | p | = | 0.553 |


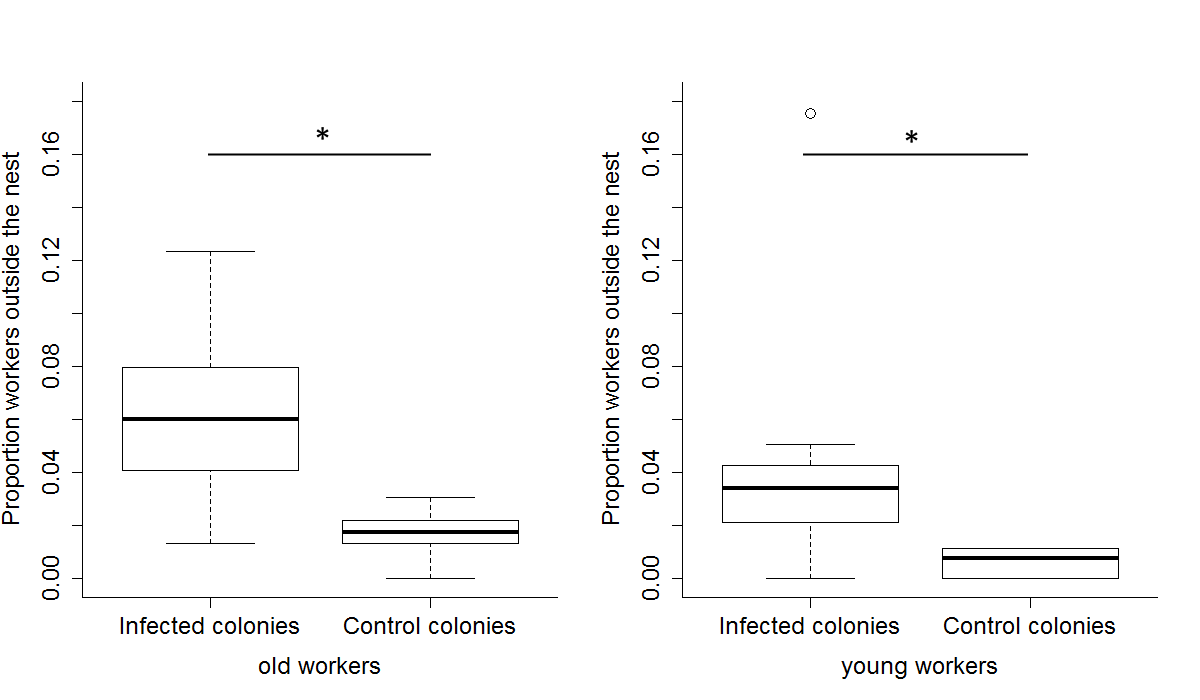


Figure S1: Proportion of old (left) and young (right) *Temnothorax crassispinus* workers leaving the nest in colonies with young workers either infected with *Metarhizium brunneum* (infected colonies) or treated with a control solution (control colonies) independent of queen presence. Both young and old workers leave the nest more often when they themselves or nestmates are infected. Boxplots show median, 25 and 75 quartile and 95% percentile (* 0.05 < p > 0.01; corrected for a false discovery rate: “fdr”).


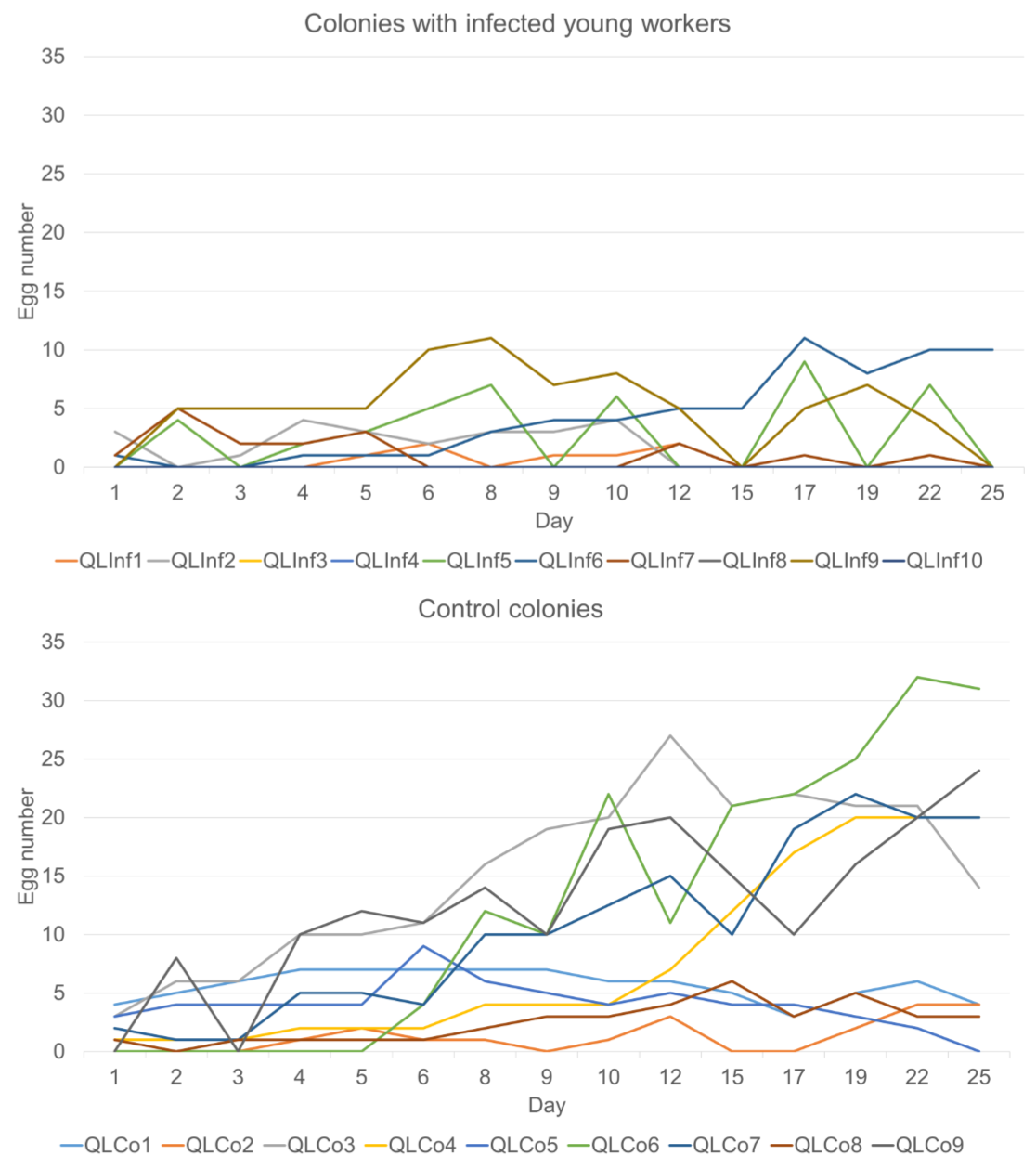


Figure S2: Reproductive rate of queenless *Temnothorax crassispinus* colonies during the first 25 days after the treatment. Eggs in infected colonies (top) vanish frequently and the colonies produce less eggs than control colonies (bottom).


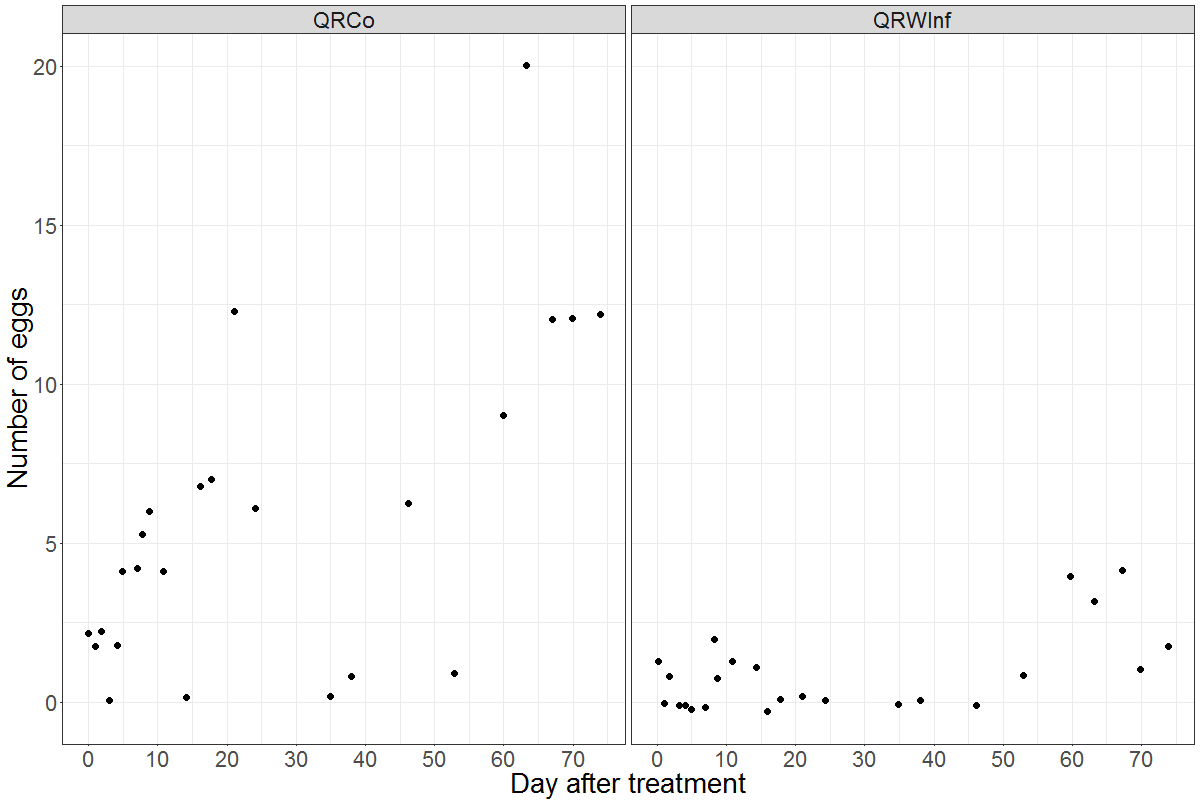


Figure S3: Number of eggs produced in queenright *T. crassispinus* control colonies (QRCo, left) or colonies with a control queen and *M. brunneum* infected workers (QRWInf, right). Whereas control queens increase their egg laying rate with time, the small number of eggs produced in colonies with infected workers vanish repeatedly.


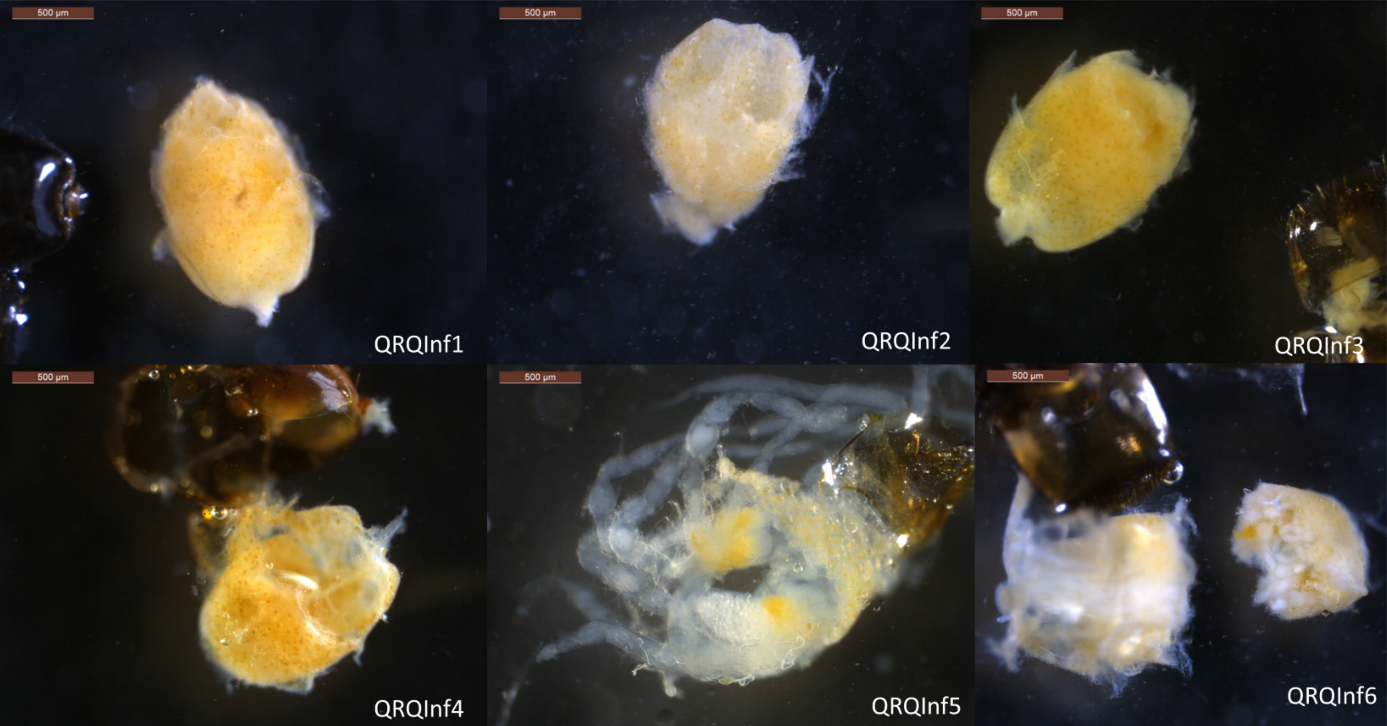


Figure S4: Ovaries of *Temnothorax crassispinus* queens infected with *Metarhizium brunneum*. Developmental status of the ovaries cannot be analyzed in five out of six queens as the gaster show excessive spore growth.
